# Sexual Health in Art and Science

**DOI:** 10.3201/eid1211.AD1211

**Published:** 2006-11

**Authors:** Salaam Semaan, Don C. Des Jarlais, Steve Bice

**Affiliations:** * Centers for Disease Control and Prevention, Atlanta, Georgia, USA;; † Beth Israel Medical Center, New York, New York, USA;; ‡ Battelle Memorial Institute, Atlanta, Georgia, USA

**Keywords:** Another Dimension, art and science, sexual behavior, Semaan, Henri de Toulouse, Paul Cézanne, Mary Cassatt, Jackson Pollock

Artists and scientists express their understanding of sexual behavior differently. Artists use visual and spatial composition; scientists use collection, analysis, and interpretation of data. However, both art and science are testaments to the creative ability of the human mind.

Scholarly work that combines art and science is often delightful. Many biomedical journals, including the Journal of the American Medical Association, Clinical Infectious Diseases, and Emerging Infectious Diseases, display images of art objects, and some relate art to health (*1*) to put a human face on the technical content. For the most part, sexual health texts use graphic illustrations to show clinical manifestations of infection and disease. Can fine art also be used to discuss sexual health?

In this article, we examine 6 art objects from the Philadelphia Museum of Art in the context of sexual health, especially the prevention and control of sexually transmitted diseases (STDs), including HIV (*2*). We combine 2 traditional approaches in our discussion of these 19th and 20th century pieces: chronology and theme (sexual health).

We begin with At the Moulin Rouge: The Dance (1890) by Henri de Toulouse-Lautrec (1864–1901) (Figure 1). Although this artist was born to an aristocratic French family, he preferred the company of bohemians. As a teenager, Toulouse-Lautrec fell twice, injuring both legs. His stunted growth was attributed to those injuries; however, more recently, doctors have blamed a rare genetic abnormality associated with dwarfism (*3*). He reached maturity with a body trunk of average size but abnormally short legs. Despite these physical limitations, he found comfort among the vivacious crowds of Paris nightclubs and brothels (*2*). Toulouse-Lautrec frequented the Moulin Rouge, a fashionable night club in the Montmartre section of Paris. Its clientele included members of the upper class, sex workers, foreign tourists, and provincial rustics (*2*).

**Figure 1:**
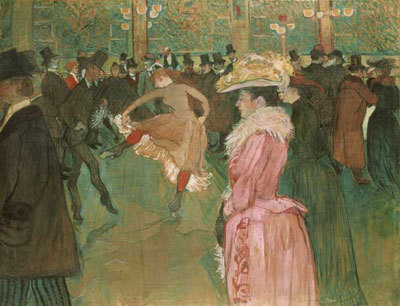
Henri de Toulouse-Latrec (1864–1901). At the Moulin Rouge: The Dance (1890). Oil on canvas (115.6 cm × 149.9 cm). The Henry P McIlhenny Collection in memory of Frances P McIlhenny, 1986. Philadelphia Museum of Art, Philadelphia, Pennsylvania, USA.

At the Moulin Rouge: The Dance portrays a mix of opposites: bright and dull colors, active dancers and passive spectators, merriment and monotonous leisure. Two dancers move energetically in the center of the canvas. The female dancer raises her skirt as she kicks out her red-stockinged legs. She gyrates so vigorously that her chignon has fallen and her skirt flares out. Her partner in a top hat is standing on tiptoe, kicking his feet as they engage in what was considered a crude, sexual dance (*4*). Surrounding the dancers is a crowd: men in top hats, a woman in a bright pink dress, other dancers, customers at the bar. Judging from her ostentatious attire and feathery hat, people at that time might have identified the woman in the pink dress as a sex worker (*2*).

In this painting are scandalous subjects: a crude dance and a sex worker (*4*). Then, as today, negative attitudes prevailed toward sex workers, who were seen as carriers of STDs (*5**,**6*). Although Toulouse-Lautrec greatly admired Edgar Degas, Degas took only passing notice of Toulouse-Lautrec, saying that some of Toulouse-Lautrec's studies of women "stank of syphilis" (*4*), which at that time was as feared as HIV/AIDS today. In Europe in the 19th century, more than 15% of the adult population and 70% of sex workers were estimated to have been infected with syphilis (*7**,**8*). Today, as then, sex workers may be viewed as immoral carriers of physical and moral hazards, including HIV/AIDS and other STDs. In turn, sex workers may mistrust healthcare providers and public health practitioners and, as a result, may not notice health messages and treatment services. Male and female sex workers may be victims of their social and economic environment, driven to sex work by poverty and lack of educational and job opportunities.

Despite his aristocratic upbringing, Toulouse-Lautrec found a way to accept and feel accepted by the entertainment industry (*4*). Sex workers were his friends, and he treated them as equals (*4*). Similarly, some public health practitioners may view sex workers as positive agents for sexual health and engage them in screening, preventive, and curative interventions for STD control and prevention.

Until the advent of penicillin in 1943, treatment for syphilis was based on the use of heavy metals such as mercury (*9*) or, as the saying goes, "a night in the arms of Venus leads to a lifetime on Mercury" (*10*). In the late 1980s, we learned that concurrent HIV infection can turn secondary syphilis back to the serious illness it was before penicillin (*11*); however, HIV-infected patients can be treated for syphilis with penicillin (*12*). At the time of Toulouse-Lautrec, who may well have contracted syphilis from 1 of his models, penicillin was not available (*4*). Shortly before his death, Toulouse-Lautrec entered a sanatorium, probably because of the adverse effects of tertiary syphilis. He died of alcoholism and syphilis at age 36 (*4*). Toulouse-Lautrec's sympathetic depiction of cabaret dancers humanized his era's sex workers. Can today's public health establishment improve the lives of HIV- and STD-infected sex workers? Today, many public health practitioners counsel sex workers about preventive and treatment services to protect them and their clients and provide them with the prospect of health and safety. Sex workers can also receive social and economic opportunities to enable them to leave the sex work industry.

While Toulouse-Lautrec painted the entertainment world, another French artist, Pierre-Auguste Renoir (1841–1919), portrayed the sensuous side of women (Figure 2). His paintings celebrated fresh air, dazzling sunlight, and pleasures of the senses (*13*). In The Great Bathers (1884–1887), Renoir shows 5 nude women bathing. Two lounge beneath a tree on a verdant riverbank while a third teasingly threatens to splash 1 of them. Further away, 2 other women frolic, seemingly indifferent to anything but the play of the hot sun and the cool fresh water on their bodies. Renoir blesses his women with luminous skin and uses color to suggest roundness. He paints them precisely, with a clean line surrounding their contours, portraying their beauty and love of life. To separate the women from the landscape, Renoir uses lemon yellows and lavenders, which create an airy image of the landscape. Translucent, bright summer light flickers on the trees and glistens on the water.

**Figure 2:**
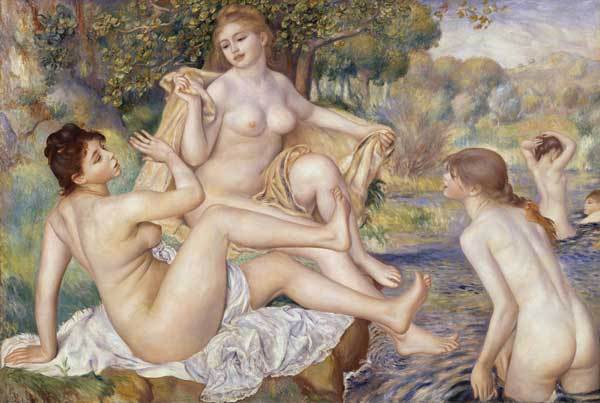
Pierre Auguste Renoir (1841–1919). The Great Bathers (1884–1887). Oil on canvas (117.8 cm × 170.8 cm) The Mr and Mrs Carroll S Tyson, Jr Collection, 1963. Philadelphia Museum of Art, Philadelphia, Pennsylvania, USA.

Apparently, Renoir did not see an ugly side to nudity; the woman in the middle of the painting was Madame Renoir. Looking at this painting, one wonders how people would behave if moral codes were not enforced and sexual infections did not exist. How did HIV/AIDS and other STDs originate? These infections take away from the pleasure of sex. In the late 1990s, it was shown that HIV-1, a retrovirus of animal origin, had probably originated from the Pan troglodytes species of chimpanzees, in which the virus coevolved over centuries (*14*). Because chimpanzees were killed for food in parts of sub-Saharan Africa, the species jump probably occurred when a hunter was exposed to the blood of an infected chimpanzee during its butchering. After the accidental transmission of the virus to humans, from infected primates and probable genetic mutations, HIV spread rapidly among population groups, facilitated by changes in global social and economic conditions (*15*).

At the time Renoir painted The Great Bathers, syphilis was prevalent and HIV epidemics did not exist (*16*). By the end of 2005, an estimated 40.3 million people worldwide were living with HIV and more than 25 million had died of AIDS (*17*). Almost 14,000 persons worldwide become infected with HIV each day, and 5 million become infected each year (*17*). The development of a safe and effective vaccine for HIV remains a formidable challenge (*16*), so safe sex is critical for disease prevention and control. As one looks at the frolicking bathers, one can vicariously enjoy their merriment and contemplate STD prevention and control, which supports human capacity for sexual intimacy within healthy relationships.

Renoir was not the only painter of nudes. Paul Cezanne (1839–1906), also a French artist, painted The Large Bathers in 1906 (Figure 3). This work portrays 14 figures with obliterated faces and truncated limbs. The ambiguity of the bathers' sex may stem from the fact that Cezanne did not use live models. He made sketches based on paintings and sculptures in museums that he later transposed to canvas.

**Figure 3:**
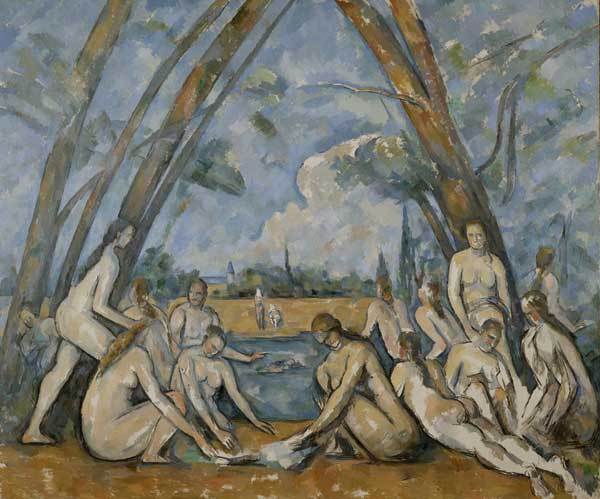
Paul Cézanne (1839–1906). The Larger Bathers (1906). Oil on canvas (210.5 cm × 250.8 cm). Purchased with the W P Wistach Fund, 1937. Philadelphia Museum of Art, Philadelphia, Pennsylvania, USA.

The 14 figures in the foreground of The Large Bathers are clustered in 2 groups, each forming a small pyramid, on each side of the painting. The figures are in an airy setting defined by refracted light and tall, slanting trees that form a pointed arch above them. Behind the figures is a person swimming. On the opposite shore appear 2 more figures. Cypress trees and a church steeple emerge from the distant wooded landscape. With somber blues, greens, and ochers, Cezanne integrates the figures into their surroundings.

How can this group of nude figures be related to sexual health? Although traditionally, individual sexual behavior has been analyzed as a determinant of HIV/AIDS and STDs, more recently, sexual mixing and sexual networks have been recognized as important mechanisms for explaining population and racial disparities in infection rates (*18*). Demographic and environmental factors create social and sexual networks that influence population-level variations in sexual behavior and infection rates of STDs and HIV. Arguably, the 14 figures could form a large sexually active group, a potentially at-risk pool for transmission of HIV/AIDS and other STDs. Public health interventions try to change peer and community norms regarding sexual health (*19*).

Concurrent sexual partnerships also explain generalized heterosexual HIV/AIDS epidemics (*20*). Serial monogamy and sporadic sexual encounters might not contribute as much to new infections as do networks of longer term concurrent or overlapping partnerships. If, for example, 1 person in a network characterized by concurrent partnerships is infected with HIV, everyone is at high risk because more people are exposed to the virus and because recently infected persons have manyfold higher viral loads and are more infectious (*21*).

Toulouse-Lautrec's painting of an entertainment hall and Renoir's and Cezanne's paintings of nude bathers show how 19th-century male artists had the liberty to congregate in unconventional venues and to paint nude figures. Contemporaneous female artists often chose more socially acceptable themes, as shown by the Maternal Kiss (1897) (Figure 4), by Mary Stevenson Cassatt (1844–1926) (*22*). This American artist left Philadelphia to study art in Paris in 1866. Because the Ecole des Beaux-Arts did not admit women, she studied with individual artists and was drawn to the group derisively called the "impressionists" (*23*). Like Renoir, Cassatt became known as a portrait painter. She focused almost exclusively on the depiction of mothers and children.

**Figure 4:**
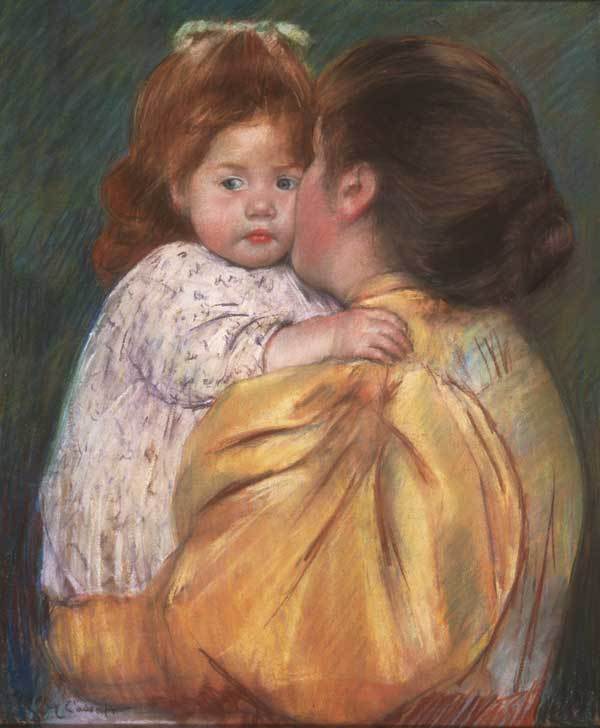
Mary Cassatt (1844–1926). Mother and Child (Maternal Kiss) (1897). Pastel on paper (55.9 cm × 45.7 cm). Bequest of Anne Hinchman, 1952. Philadelphia Museum of Art, Philadelphia, Pennsylvania, USA.

Maternal Kiss portrays an intimate and tender moment between a mother and child. The child forms the psychological focus of the painting as the mother's features are lost in the child's cheek. The painting displays a subtle richness of color in the iridescent salmon-hued leg-of-mutton sleeve of the mother's dress and the delicate fabric clothing of the auburn-haired baby.

In an ideal world, mothers pass on to their children their love and wisdom; however, mothers infected with HIV or other viral STDs can pass these infections to their babies (*24**,**25*). An infant can acquire HIV infection during pregnancy, labor, delivery, or breastfeeding (*24*). Although perinatal HIV infections in the United States peaked in 1991 at an estimated 1,650, they declined in 2002 to an estimated 144–236 (*24*). Preventive and curative interventions have reduced perinatal HIV transmission in the United States to less than 2%, compared with 25%–30% without such interventions (*26*). Effective interventions include routine HIV screening of pregnant women, use of antiretroviral drugs for treatment and prophylaxis, avoidance of breastfeeding when the mother is HIV-infected, and use of elective cesarean delivery when appropriate. However, approximately 1,800 HIV-infected infants are born each day worldwide, most of them in sub-Saharan Africa (*27*). In 2003, an estimated 700,000 new HIV infections occurred in children worldwide—almost all from mother-to-child transmission.

At the time of Cassatt, congenital syphilis was a major concern because it caused miscarriages and stillbirths (*28*). A tragic possibility is that even today, a baby could be spared HIV infection, only to a die a few weeks later of congenital syphilis (*29*), as has been reported recently in several countries (*30**,**31*).

Marcel Duchamp (1887–1968), an American artist, born in France, was a scion of an artistic family. His Given (image not shown) (also known as Etant Donnes: 1. la chute d'eau, 2. le gaz d'eclairage [Given: 1. The Waterfall, 2. The Illuminating Gas]) (image not shown) is a unique example of an art installation and presents a complex narrative in a multimedia format. Given (1946–1966) shows a naked woman behind a closed door. In the center of roughly stuccoed wall is a large arched doorway made of old bricks (*32*). The door is weathered silver gray, studded with iron rivets, and shows no sign of hinges, knob, or handle, confirming the impression that the door cannot be opened. In the middle of the door, at eye level, 2 small holes invite inspection of the 3-dimensional tableau that lies behind. As the viewer steps onto a mat in front of the door, the lights become activated so the viewer can peer through the holes for a private experience of what is within. Gazing through several layers of space, the viewer sees a nude woman lying on her back among a mass of twigs and leaves. Her face is farthest away and hidden by a wave of blonde hair. Her legs are spread and extend toward the door; her feet are obscured by the brick wall. Her right arm cannot be seen, but her left arm is raised, holding in her hand the glass fixture of a small gas lamp that glows faintly. In the distance is a hilly, wooded landscape that rises above a pond. Clouds are soft and white in the blue sky. To the far right is a waterfall.

Duchamp's installation can be disturbing, as one is suddenly confronted with an unexpected and shockingly graphic image of a naked woman behind the door. In this installation, Duchamp has determined forever the exact amount of detail and the fixed perspective he intended for the viewer. One is unable to walk around Given, to get closer to peer at details, or to back away for a different perspective. Similarly, talking about sex, even in the context of prevention and control of infection, can be disturbing. Because sex is a private matter, to optimize prevention and treatment, scientists and healthcare providers depend on the information provided by research participants and patients. Accurate reporting is crucial for treating patients and their sex partners, for monitoring trends of sexual behavior and infection rates, and for prevention and treatment. Inaccurate reporting can distort clinical decisions; can compromise diagnostic, preventive, and therapeutic interventions; and can hinder partner notification and referral services. Therefore, public health practitioners and healthcare providers strive for rapport and trust with research participants and patients.

Because sexual behavior is influenced by personal and societal attitudes, reporting and sharing of information is often subject to reporting bias, which arises when people do not reveal private information, even for health reasons. This type of bias is referred to as "social desirability bias" because what is considered socially desirable or undesirable behavior affects whether a person reports it accurately. Studies show that reporting of sexual behavior and infection status can be inaccurate, even when such information is shared with healthcare providers (*33*). Just as Duchamp challenges and disturbs the viewer, patients often challenge public health practitioners and healthcare providers. Successful public health interventions must overcome the uncomfortable aspects of sexual health communication.

The validity of reported data about sexual health can be enhanced. Procedures and laws protect people's privacy and the confidentiality and security of collected data (*34*). To maximize self-report accuracy, investigators ask respondents to provide information on recent sexual behavior with short recall times, e.g., "over the past 4 weeks" rather than "over the past 2 years" (*35*). Investigators also administer questionnaires in a confidential manner. They use self-administered questionnaires and computer-assisted technology, such as audio or telephone computer-assisted self-interviewing (*36**,**37*). Biologic markers are often used to ascertain validity of reported data (*38*).

With Duchamp's Given, we noted the importance of accurate reporting of sexual behavior. To note the importance of communication between partners, we explored Jackson Pollock's Male and Female (1942) (Figure 5). Pollock (1912–1956), an American artist born in Wyoming, earned a reputation for his classic drip paintings. He was instrumental in creating a new concept of art in which exuberant energy and motion were made visible (*39*). Pollock created his paintings on the floor rather on an easel, thereby enabling him to use his entire body to pour paint on the canvas. Pollock believed that artists did not need to go outside themselves for subject matter. He advocated that artists tap the unconscious mind, an art perspective that came to be known as abstract expressionism (*40*).

**Figure 5:**
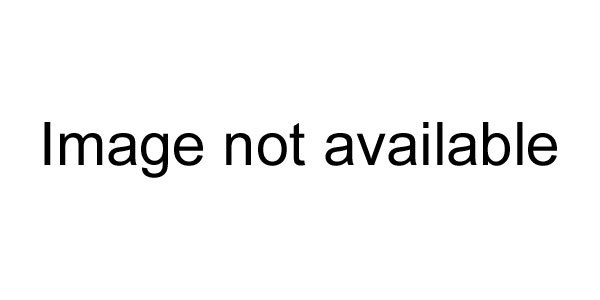
Jackson Pollock (1912–1956). Male and Female (1942). Oil on canvas (186.1 cm × 124.3 cm). Gift of Mr and Mrs H Gates Lloyd, 1974. Philadelphia Museum of Art, Philadelphia, Pennsylvania, USA. Licensed by Artists Rights Society, New York, New York, USA.

Male and Female engages the viewer quickly because of its vibrant colors and emotional brushwork (*2*). The painting is characterized by skeins of dripping paint and by scratching and scraping that expose the canvas. The painting consists of 2 centrally placed, youthful figures. While the eyelashes and curvaceous forms of the figure on the left and the more angular form and numbers on the figure on the right predispose one to assume that the former is female and the latter is male, the sex of each figure remains ambiguous. The figures can be seen as facing each other, both facing to the left, or both turning their backs to each other. The figures stand in the midst of a complex network of signs, numbers, and splattered paint.

Was Pollock possibly portraying the complex communication patterns between the sexes? Communication, defined as exchange of information, is key to interactions and sexual health. However, despite being crucial for preventing HIV infection and other STDs, communication about sexual health has always been emotionally charged (*41*). Different factors affect women's communication about sexual health or the use of condoms with their male partners. Most commonly reported are guilt and shame, fear of personal violence, abandonment, economic repercussions, and harsh judgment (*42**,**43*). Cultural expectations to be passive make it more difficult for women to take responsibility for their sexual health and prepare for possible sexual encounters (*44*). A broader spectrum of behavioral skills and biomedical interventions, such as microbicides, offers alternatives to avoidance of risk-producing situations (*45*). Nevertheless, sex partners still need to talk about safe sex, encourage mutual testing for HIV and other STDs, and discuss test outcomes and preventive and treatment regimens.

Did Pollock intend Male and Female to teach about sexual health communication? HIV risk-reduction interventions teach communication skills as an important component in prevention and control of HIV and other STDs (*46*). These interventions seek to change behavior by providing information about risk reduction, partner communication, sexual assertiveness, sexual negotiation, refusal to have unsafe sex, and avoiding or minimizing partner abuse and violence during disclosure of STDs (*47*).

Communication about sexual health is also important between parents and children. Many parents find it difficult to talk with their children about safe sex. One father jokingly remarked that he believed in making the world safe for his children, but not for his children's children because he did not think his children should have sex or talk about sex. Educational interventions can help parents feel more comfortable talking with their children about sexual health (*48*). Some believe that communication about sexual health can have adverse behavioral outcomes, such as increased sexual activity, risky sexual behavior, or earlier sexual debut. However, HIV interventions to change risk behavior have not been associated with unintended negative consequences (*49*).

Duchamp hid the nude woman behind closed doors, and possibly, Pollock portrayed the communication dynamics between the sexes, expressing social expectations about sexual behavior and health. Because sex and sexuality are sensitive subjects, policymakers hesitate to discuss them. But leadership in policy and science is needed to prevent and control transmission of infections. The spread of HIV has necessitated discussion of sexual behavior and health to promote preventive behavior and connect people with appropriate care. Breaking the silence and stigma that surround sexual behavior communication enhances sexual health (*50*). Without addressing societal barriers, prevention and treatment interventions cannot achieve their full potential.

Scientists and artists examine and portray the pleasure and pain of sexual intimacy and sexual health. Artists portray human sexuality on canvas, in sculpture, and through art installations. They use various techniques, portray different degrees of sensuality, and evoke multiple emotions. When artists deal with human sexuality, we can learn about sexual health. This bridging process is needed because discussing sexual health is challenging, given the private nature of sexual behavior, the social stigma associated with many sexual practices and with HIV/AIDS and STDs, and the moral values associated with sexual behavior. Fighting silence and stigma and promoting empowered relationships can control infection. Addressing social factors that facilitate transmission of STDs and HIV/AIDS and advocating for strong leadership are necessary.

Medical illustrations, as used in textbooks, depict clinical manifestations of disease to teach about prevention and treatment. However, fine art can provide useful starting points for teaching and generating discussion. Art and science remind us of the joy and pain of human intimacy, the need for responsible sexual behavior, and the importance of prevention and control of HIV/AIDS and other STDs.

## Appendix

Dr Semaan is acting associate director for science of the National Center for HIV, Viral Hepatitis, STD, and TB Prevention (proposed) at the Centers for Disease Control and Prevention. After completing her docent training at the Philadelphia Museum of Art and at the High Museum of Art in Atlanta, she served as a volunteer museum tour guide. She was inspired to write this article after she gave a guided tour of the Philadelphia Museum of Art to attendees of the 2003 National STD Prevention Conference.

Dr Des Jarlais is director of research at Baron Edmond de Rothschild Chemical Dependency Institute of the Beth Israel Medical Center in New York City. When he was 6 years old and living in Germany, his parents began to drag him to art museums throughout Europe. Despite his protests at the time, this led to a lifelong interest in art. Living in New York City and frequently traveling internationally provide him with many opportunities to visit art museums and exhibits. Dr Des Jarlais' effort on this paper was supported by grants DA 03574 and DA 11041 from the National Institutes of Health.

Mr Bice is CDC's former director of the Division for the Strategic National Stockpile and the current senior manager for life sciences at Battelle Memorial Institute, Atlanta. His work covers the prevention and control of emerging infectious diseases and emergency preparedness, including response and medical logistics. During his undergraduate work, Mr. Bice took courses in the fields of art history and in commercial and industrial design. His interest in the arts continues to this day.

**Additional Resources**

1. Aral SO. Understanding racial-ethnic and societal differentials in STI. Sex Transm Infect. 2002;78:2–4. 10.1136/sti.78.1.211872846PMC1763713

2. Des Jarlais DC, Semaan S. HIV prevention research: cumulative knowledge or accumulating studies? J Acquir Immune Defic Syndr. 2002;30(Suppl 1):S1–7.12107355

3. Karcioglu ZA. Ocular pathology in the parable of the blind leading the blind and other paintings by Pieter Bruegel. Surv Ophthalmol. 2002;47:55–62. 10.1016/S0039-6257(01)00290-911801271

4. Potts M, Short R. Ever since Adam and Eve: the evolution of human sexuality. Cambridge (UK): Cambridge University Press; 1999.
